# Cerebral Autoregulation: Don’t go with the Flow, be the Flow; Author′s Response

**DOI:** 10.1007/s12028-023-01759-5

**Published:** 2023-06-08

**Authors:** Björn B. Hofmann, Christian Rubbert, Kerim Beseoglu

**Affiliations:** 1 Department of Neurosurgery, Medical Faculty and University Hospital Düsseldorf, Heinrich-Heine-University, Düsseldorf, Germany; 2 Department of Diagnostic and Interventional Radiology, Medical Faculty and University Hospital Düsseldorf, Heinrich-Heine-University, Düsseldorf, Germany

Dear editor,

We would like to thank Ayasse et al. for their interest in our article “Blood Pressure Affects the Early CT Perfusion Imaging in Patients with aSAH Reflecting Early Disturbed Autoregulation” [1] and for providing their insights into cerebral autoregulation. We appreciate the opportunity to respond to the issues raised.

Firstly, we would like to emphasize that our study primarily focused on the correlation between mean transit time (MTT) of early computed tomography perfusion (CTP) imaging and blood pressure during the early brain injury (EBI) phase, indeed on a population level.

As Ayasse et al. alluded to, MTT was chosen as the parameter of interest because it is known to be the most relevant prognostic CTP parameter in patients with aneurysmal subarachnoid hemorrhage (aSAH), and a shorter MTT is associated with better outcomes and fewer complications [2–4]. Neither cerebral blood volume (CBV) nor cerebral blood flow (CBF) was shown to be a relevant factor in that regard. However, the quotient of these two parameters, the MTT, was found to apparently better reflect the relevant pathophysiology radiologically in the EBI phase in a clinical setting [2–4].

In part, this may be explained by the relative nature of CBV and CBF measurements in CTP imaging, and we agree with the statement by Ayasse et al. that it is essential to carefully choose the method for quantitatively establishing CBF. Both CBV and CBF measurements include a measure of mass of brain tissue in the respective units, which is an unknown during a CTP scan, thereby limiting comparability across patients and even across different imaging time points in the same patient. In MTT, however, as the quotient of CBV/CBF, all relative units are reduced from the results without any elaborate postprocessing, leaving only an absolute measurement: time. We acknowledge that CBF and CBV measurements are abundant in the literature, and different evaluation methods and software lead to variability in reported MTT values. However, when trying to find suitable surrogate parameters with potential for real-world clinical usage, we try to concentrate on “more comparable” parameters, such as MTT, where possible.

Following the concepts of cerebral autoregulation laid out by Ayasse et al., a prolongation of MTT is indeed to be expected in patients with a decreased MAP, and we thank Ayasse et al. for contributing the valuable and clear explanations of the relationship between MAP, CBF, CBV, and MTT. Although both our title and the conclusion are arguably pointed, we feel that our discussion is a bit more nuanced and better explains our conclusions. Although CBV and CBF were not considered in our study, as explained previously, it has to be noted that at least some earlier observations have reported reduced CBF and constant CBV in CTP imaging in the EBI phase [3, 4] and that other studies have described impaired autoregulation in the EBI phase [5]. Instead, we would like to draw the reader’s attention to the increasingly stronger correlation between MAP and MTT as the patient’s neurological status worsens, as measured by the World Federation of Neurosurgical Societies grade. We found it remarkable to demonstrate, for the first time, that in less vigilant patients (a direct indicator of the severity of EBI), the correlation between MAP and MTT becomes stronger. We assumed that the most probable cause for this observation was the impairment of cerebral autoregulation due to the severity of EBI. We appreciate that our thought process should have been laid out more carefully, and we are thankful to Ayasse et al. for providing this opportunity.

We remain convinced that disturbances in cerebral autoregulation play an important role in the EBI phase in patients with aSAH, and further efforts should be made to understand these relationships, especially in patients with poor-grade aSAH. Finally, we would like to extend our gratitude to Ayasse et al. for contributing to the discussion with clear and constructive comments. We hope that our response clarifies the issues raised in the letter and provides additional insight into the complexity of this topic.
